# Expanded identification and characterization of mammalian circular RNAs

**DOI:** 10.1186/s13059-014-0409-z

**Published:** 2014-07-29

**Authors:** Junjie U Guo, Vikram Agarwal, Huili Guo, David P Bartel

**Affiliations:** Whitehead Institute for Biomedical Research, Cambridge, MA 02142 USA; Howard Hughes Medical Institute, Chevy Chase, MD 20815 USA; Department of Biology, Massachusetts Institute of Technology, Cambridge, MA 02139 USA; Computational and Systems Biology Program, Massachusetts Institute of Technology, Cambridge, MA 02139 USA; Current address: Institute of Molecular and Cell Biology, Singapore, 138673 Singapore; Current address: Department of Biological Sciences, National University of Singapore, Singapore, 117543 Singapore; Current address: Lee Kong Chian School of Medicine, Nanyang Technological University-Imperial College, Singapore, 639798 Singapore

## Abstract

**Background:**

The recent reports of two circular RNAs (circRNAs) with strong potential to act as microRNA (miRNA) sponges suggest that circRNAs might play important roles in regulating gene expression. However, the global properties of circRNAs are not well understood.

**Results:**

We developed a computational pipeline to identify circRNAs and quantify their relative abundance from RNA-seq data. Applying this pipeline to a large set of non-poly(A)-selected RNA-seq data from the ENCODE project, we annotated 7,112 human circRNAs that were estimated to comprise at least 10% of the transcripts accumulating from their loci. Most circRNAs are expressed in only a few cell types and at low abundance, but they are no more cell-type-specific than are mRNAs with similar overall expression levels. Although most circRNAs overlap protein-coding sequences, ribosome profiling provides no evidence for their translation. We also annotated 635 mouse circRNAs, and although 20% of them are orthologous to human circRNAs, the sequence conservation of these circRNA orthologs is no higher than that of their neighboring linear exons. The previously proposed miR-7 sponge, CDR1as, is one of only two circRNAs with more miRNA sites than expected by chance, with the next best miRNA-sponge candidate deriving from a gene encoding a primate-specific zinc-finger protein, ZNF91.

**Conclusions:**

Our results provide a new framework for future investigation of this intriguing topological isoform while raising doubts regarding a biological function of most circRNAs.

**Electronic supplementary material:**

The online version of this article (doi:10.1186/s13059-014-0409-z) contains supplementary material, which is available to authorized users.

## Background

Many classes of non-protein-coding RNAs (ncRNAs) exist in cells [1,2], and members of each class play important roles in either regulating gene expression or other biological processes [3–6]. For example, microRNAs (miRNAs) pair to sites within messenger RNAs (mRNAs) to target the mRNAs for translational repression and/or mRNA destabilization [7]. In an intriguing elaboration of this regulatory pathway, the activity of the mammalian miR-7 miRNA can be inhibited by CDR1as/ciRS-7, which is in turn targeted by another miRNA, miR-671, which shows near-perfect complementarity and triggers endonucleolytic cleavage of CDR1as [8–10]. CDR1as is a circular RNA (circRNA) deriving from an antisense transcript of the CDR1 protein-coding gene [10]. With >60 conserved sites for miR-7, CDR1as is thought to act as a sponge to titrate miR-7 from its other targets [8,9]. A second circRNA proposed to act as a sponge is the testis-specific transcript of the male sex-determining gene *Sry*, which contains 16 sites for miR-138 [9]. Because circRNAs lack poly(A) tails and 5′ termini, they would escape the deadenylation, decapping and degradation normally caused by miRNA association [11], an obvious advantage for an RNA acting as a miRNA sponge [8,9].

**Figure 1:**
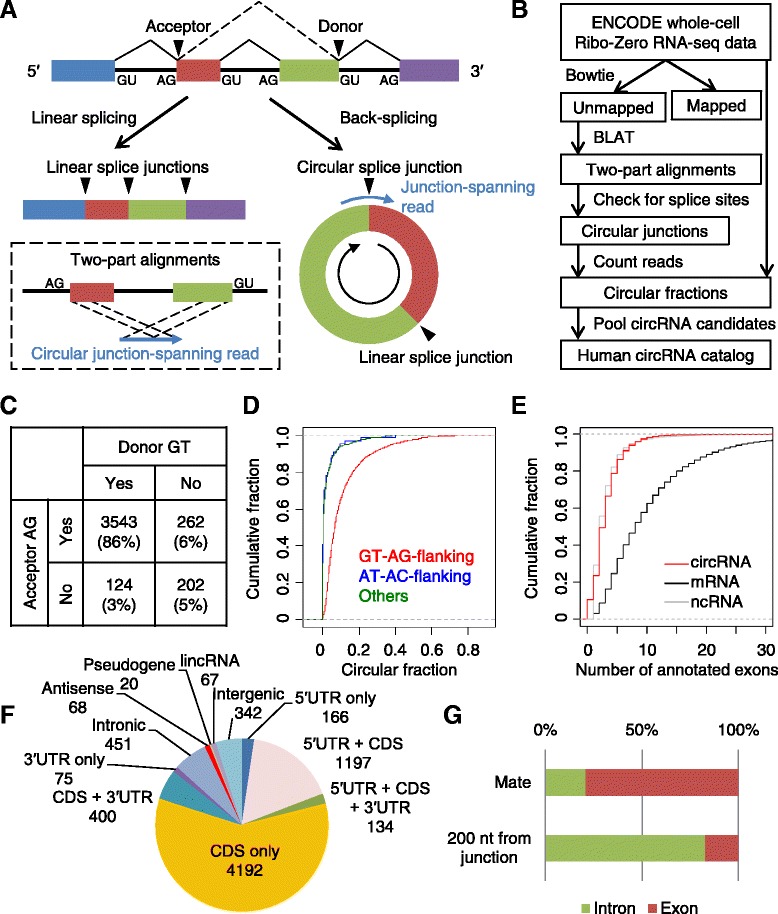
**Global identification of human circRNAs. (A)** Schematic illustration of the alternative-splicing isoforms generated from linear splicing (left) and back splicing (right). Two-part alignments identified junction-spanning reads indicative of circRNAs (bottom left). Exons are colored, and donor (GU) and acceptor (AG) signals at splice sites are indicated. **(B)** The computational pipeline developed to identify and quantify circRNAs from long-read RNA-seq data. **(C)** Enrichment of donor GT and acceptor AG splicing signals in genomic windows flanking candidate circular junctions supported by ≥5 junction-spanning reads in the CD34 sample. Similar results were obtained from all other cell types. **(D)** Distribution of circular fractions for circRNA candidates in (C), grouped based on whether their circular junctions were flanked by splicing signals of the major or minor spliceosome (GT-AG- and AT-AC-flanking, respectively). **(E)** Distributions of exon numbers for circRNAs, mRNAs, and other annotated ncRNAs. **(F)** Annotations of genomic regions mapping to inferred circRNA exons. CDS, coding sequence; lincRNA, long intervening ncRNA; UTR, untranslated region. **(G)** Splicing within circRNAs of the CD34 sample. Mapped locations of the mates of junction-spanning reads were compared to the genomic annotations 200 nucleotides downstream and upstream of back-spliced acceptors and donors, respectively. Because the fragment size for the paired-end sequencing averaged 200 nucleotides, these genomic annotations resembled those expected if the introns within the circRNAs were retained.

Thousands of additional circRNAs with unknown functions have been identified in various species [8,12–15]. These circRNAs are generated primarily through a type of alternative RNA splicing called ‘back-splicing’, in which a splice donor splices to an upstream acceptor rather than a downstream acceptor (Figure 1A) [8,12,14,16,17]. Based on several criteria, including their intriguing expression patterns, their apparently elevated sequence conservation and the compelling hypothesis that CDR1as acts as a miR-7 sponge, these circRNAs have been proposed to comprise a large class of post-transcriptional regulators. However, the number of additional circRNAs acting as natural miRNA sponges is currently unclear. Indeed, the extent to which these circular isoforms might act in any biological capacity is not known.

To begin to consider potential roles of circRNAs in post-transcriptional regulation, we developed a computational pipeline that identifies circRNAs from long-read RNA-seq data without relying on gene annotations. The pipeline resembled that reported previously [8], except it quantifies and considers the abundance of each circular isoform with respect to its alternative linear isoforms. Applying this pipeline to the non-poly(A)-selected RNA-seq data from the ENCODE project, we catalogued >7,000 human circRNAs and characterized their global properties, acquiring new insights regarding their biogenesis, the cell-type specificity of their expression, the extent to which they are conserved, the extent to which they are translated and their potential to act as miRNA sponges.

## Results

### Properties of human circRNAs

To identify circRNAs from RNA-seq data, we developed the following computational pipeline (Figure 1B). We first mapped all the RNA-seq reads to the genome using Bowtie in single-end mode, allowing ≤2 mismatches. Then we used BLAT to find partial alignment of the unmapped reads. Dual alignments of two read segments mapping to the genome in the reversed order were indicative of circRNAs. The circular fraction (that is, the fraction of the circular isoform relative to all transcripts from the same locus) was quantified for each circRNA candidate by counting relevant reads from the same sample. We performed circRNA identification and quantification using all the currently available whole-cell non-poly(A)-selected RNA-seq data from the ENCODE project [1], which included a large variety of cultured cell types (Table S1A in Additional file 1). As in some previous studies [8,14], our pipeline used the assembled genome for sequence alignment but disregarded its annotations, and thus it was not affected by incomplete or inaccurate genome annotations and was not biased in favor of alternative isoforms of pre-mRNAs.

circRNAs produced from back-splicing would be expected to have splicing signals at their junctions. Introns spliced by the major spliceosome usually contain the GU dinucleotide at their 5′ end (the splice donor) and the AG dinucleotide at their 3′ end (the splice acceptor) [18]. Indeed, when we analyzed all the dinucleotide frequencies in 10-nucleotide genomic windows mapping to each observed circular junction, a vast majority of candidate circular junctions contained the GT dinucleotide within 5 nucleotides of the putative donor end and the AG dinucleotide within 5 nucleotides of the putative acceptor end (Figure 1C; Figure S1A in Additional file 2). Moreover, a search for motifs within 10-nucleotide genomic windows flanking the circular junctions recaptured the canonical sequence motifs of splice donors and acceptors (Figure S1B in Additional file 2). When considering the minority of candidates without GT-AG-flanking junctions, no pronounced dinucleotide enrichment or significant motif was observed (Figure S1A,B in Additional file 2).

Reasoning that for biological circRNAs a higher fraction of the transcript isoforms might be circular, as is the case for CDR1as, for which almost no linear isoform could be detected [8,9], we calculated for each candidate the fraction of its transcript isoforms that were circular and compared the circular fractions of groups of circRNA candidates with different flanking dinucleotide signatures. The circular fractions of GT-AG-flanking candidates tended to be greater than those of the remaining candidates, with the circular fractions of most non-GT-AG-flanking candidates falling below 1% (Figure 1D). To test the extent to which the minor spliceosome might contribute to circRNA formation, we examined the distribution of circular fractions for AT-AC-flanking candidates, but observed no difference from the other non-GT-AG-flanking candidates (Figure 1D).

Collectively, these results indicated that back-splicing by the major spliceosome generates most, if not all, cellular circRNAs. Candidates without these splicing signals were more likely to have arisen from sequencing artifacts (such as chimeric RNA-seq reads resulting from template switching during reverse transcription or PCR), which justified the filter for GT-AG splicing signals imposed in previous pipelines [8]. To maximize the specificity of our pipeline, we carried forward only those candidates flanked by the GT-AG splicing signals, recognizing the possibility that a few candidates discarded by this filter might be authentic circRNAs generated by mechanisms that do not involve the spliceosome, as shown in Archaea [13]. As a second quality filter, we also required that each circRNA have a circular fraction ≥10% in two or more samples. This requirement filtered out about two-thirds of the circRNAs in each sample. With these filters, we annotated 7,112 circRNAs from 39 biological samples representing a large variety of human cell lines (Table S2A in Additional file 3).

Assuming that each circRNA had the same exon structure as the current GENCODE annotation at its locus, we found that most circRNAs spanned <5 exons (Figure 1E), with the distribution of exon abundance resembling that reported for the other GENCODE-annotated ncRNA genes in the human genome [2]. The distribution of circRNA exonic sequence lengths also resembled that of ncRNAs, with a median length of 547 nucleotides, compared with 566 and 2,149 nucleotides for ncRNAs and mRNAs, respectively (Additional file 4). More than half of the circRNAs consisted of only protein-coding exons (Figure 1F), whereas smaller fractions also contained 5′ untranslated regions (UTRs), 3′ UTRs, or both. CDR1as was among the 68 circRNAs that mapped antisense to annotated protein-coding genes. Another 67 circRNAs mapped to annotated long intervening ncRNAs (lincRNAs) [19], and 342 mapped between annotated genes, with no sense or antisense overlap.

Because many circRNAs contained multiple exons (Figure 1E) and previous studies have noticed retained introns in a few circRNAs [10,15], we more systematically examined whether introns within circRNAs were efficiently removed. We started by mapping all the mate reads of the circular junction-spanning reads in the CD34^+^ hematopoietic progenitor cells sample. If intra-circular splicing did not occur, most of the mate reads would be expected to map to the first upstream or downstream intron from the back-spliced donor or acceptor, respectively (Figure 1G). We found that approximately 80% of the mates reads that did not map to the same exons as the circular junctions mapped to their neighboring exons, indicating that introns within circRNAs were usually spliced out, although a substantial fraction (approximately 20%) were retained (Figure 1G).

### Comparison with previous circRNA catalogs

When comparing our circRNA catalog with those of previous studies, we found that most annotated circRNAs were present in only one catalog (Additional file 5), presumably because of differences in cell types, cutoffs and computational pipelines. A key difference between our catalog and those of others was our requirement that the circRNAs have a circular fraction ≥10%, which prompted us to examine the extent to which this filter explained the differences between our catalog and those of others. For each catalog, we randomly selected one cell type used to build the catalog and quantified the circular fraction of the circRNAs identified in that cell type by the corresponding study, using non-poly(A)-selected RNA-seq data of that cell type. Due to our circular-fraction filter, all the circRNAs from our study had circular fractions of ≥10% (Additional file 5). About half of the circRNAs identified by the Memczak *et al*. study [8] had circular fractions of ≥10%, whereas less than 10% of the circRNAs from the other two studies, which used either RNase R-treated [14] or poly(A)-depleted RNA-seq data [15] to enrich for circRNAs, had circular fractions ≥10%.

### Trans-splicing rarely contributed to back-spliced junctions

**Figure 2:**
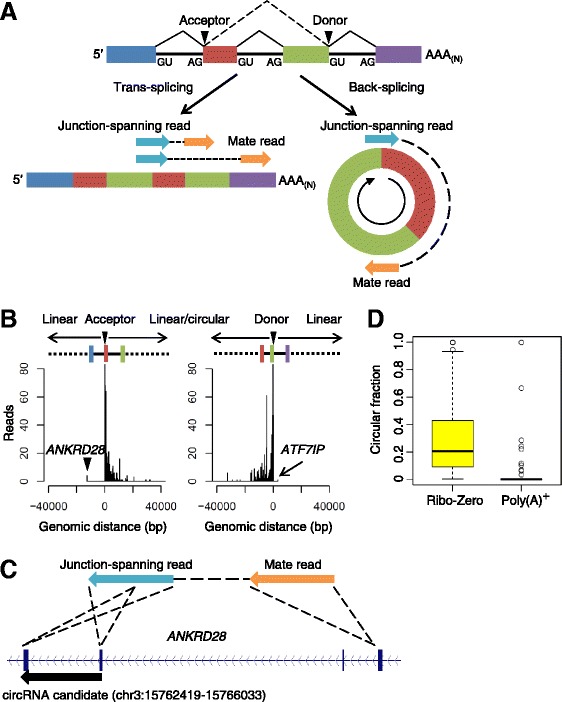
**Trans-splicing rarely contributed to back-spliced junctions. (A)** Schematic illustration of the analysis of paired-end reads used to distinguish trans-spliced products from circRNAs. Depending on the insert size, mate reads of trans-spliced but not back-spliced junction-spanning reads could potentially map to adjacent linear exons. Based on the insert sizes of the ENCODE paired-end RNA-seq libraries, we only considered circRNAs that were <400 nucleotides. **(B)** Distances of all mapped mate reads from the acceptors (left) and donors (right). Two possible trans-spliced events are indicated. **(C)** The identified trans-spliced event from the *ANKRD28* locus. **(D)** Circular fractions of 598 circRNAs detected in non-poly(A)-selected RNA-seq data from U2OS cells, analyzed using non-poly(A)-selected RNA-seq data (Ribo-Zero) and poly(A)-selected RNA-seq data (poly(A)^+^).

Trans-splicing between pre-mRNAs can also give rise to the appearance of shuffled exons [20,21], many of which would produce sequencing reads indistinguishable from those that we and others [8] attributed to back-spliced products (Figure 2A). To distinguish between back-splicing and trans-splicing, we used the approach used previously on a smaller set of circRNAs [12]. This approach took advantage of the paired-end RNA-seq data and examined the mate reads of the junction-spanning reads, which for some trans-spliced products would map beyond the genomic regions spanning the acceptors and donors of the junction-spanning reads (Figure 2A). Out of >6,000 mates of junction-spanning reads mapped in the CD34^+^ hematopoietic progenitor cells sample, only four (all from the *ANKRD28* locus) mapped upstream of the back-spliced acceptors, and only one (from the *ATF7IP* locus) mapped downstream of the back-spliced donors (Figure 2B,C).

Although analysis of mate reads would have identified more trans-spliced products if many members of our catalog were in fact trans-spliced and not circular, this analysis presumably missed evidence of trans-splicing in cases for which the exonic distance between the trans-spliced acceptor and donor was too large to exclude any mate reads, which was the case for most circRNAs (Additional file 4). As an orthogonal approach for discriminating between back-spliced and trans-spliced products we considered their polyadenylation status [12]. Poly(A) selection should deplete circRNAs but not trans-spliced products, which are linear and thus expected to have poly(A) tails (Figure 2A). Indeed, using data from U2OS cells, which were independent of the data we used for circRNA discovery, we found that of the 598 members of our catalog detected through junction-spanning reads in non-poly(A)-selected RNA-seq data, only 20 were detected in poly(A)-selected RNA-seq data, as indicated by circular fractions exceeding zero for only these 20 members in the poly(A)-selected data (Figure 2D). Moreover, only six members of our catalog were detected in the poly(A)-selected data but not the non-poly(A)-selected data. The 20 detected in both datasets presumably include both trans-spliced products and circRNAs from loci that also produce trans-spliced isoforms. These observations, in conjunction with the lack of translation across the circular junctions (see below), indicated that trans-splicing contributed very few (<5%) false positives in our circRNA catalog, despite a previous study reporting that shuffled splice isoforms are predominantly trans-spliced products [20]. We attribute our high specificity to our use of non-poly(A)-selected samples for circRNA identification (whereas the previous report started with poly(A)-selected samples) and our requirement that the circular fraction exceeded 10% in at least two samples. These results are consistent with previous studies showing that circRNAs are non-polyadenylated [12] or RNase R-resistant [8,14].

### Expression of circRNAs

**Figure 3:**
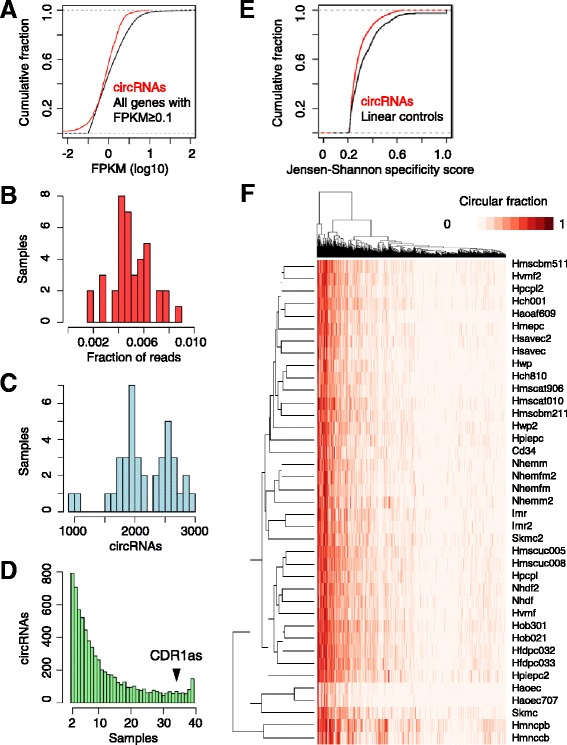
**Expression of human circRNAs. (A)** Levels of circRNAs in CD34^+^ hematopoietic progenitor cells. The expression level was estimated for each circRNA (using its circular fraction and the FPKM of the corresponding gene, which included both circular and linear isoforms) and the cumulative distribution of levels is plotted. For comparison, the levels of mRNAs with FPKM ≥0.1 are also plotted. **(B)** Fractions of mRNA-mapping reads estimated to derive from circRNAs. Reads derived from each circRNA were estimated as the product of the circular fraction, the gene FPKM and the length of the circRNA exonic sequence. The fraction was estimated for each sample, and the distribution of fractions is plotted. **(C)** Numbers of circRNAs identified in each biological sample. The number of circRNAs was tallied for each sample, and the distribution of values is plotted. **(D)** Numbers of samples in which ≥10% circular fraction was observed. The number of samples with ≥10% circular fraction was tallied for each circRNA, and the distribution of values is plotted. **(E)** Cumulative distribution of cell-type-specificity scores of circRNAs compared to mRNAs with similar overall expression levels (linear controls). **(F)** Unsupervised hierarchical clustering of the circular fractions of 1,299 circRNAs for which the availability of both the donor and the acceptor sites were each supported by ≥5 reads in all 39 samples.

To act as miRNA sponges or perform other non-catalytic cellular functions, the circRNAs would need to be expressed at consequential levels within the cell. To infer the abundance of each circRNA we multiplied its circular fraction by the density of RNA-seq reads arising from the cognate gene locus (measured in fragments per kilobase of transcript per million fragments sequenced, or FPKM). As observed for all protein-coding genes with FPKM ≥0.1, approximately 40% of all circRNAs annotated from each cell type had an inferred FPKM ≥1, as illustrated for the CD34^+^ hematopoietic progenitor cells sample (Figure 3A). However, the abundances of circRNAs tailed off much more quickly than did those of mRNAs. For example, when considering the 562 circRNAs with inferred FPKM ≥1.0, only 37 had FPKM ≥10 and none had FPKM ≥100. As a result, our circRNAs comprised a small fraction of the transcriptome of each sample, accounting for an estimated 0.2 to 0.9% of all the exon-mapping reads (Figure 3B). This range is slightly lower than a recent estimate of 1% [15], presumably because most low circular-fraction circRNAs were discarded in our analysis.

We next examined the cell type specificity of circRNA expression. The 39 biological samples varied in the number of detectable circRNAs (Figure 3C). Although 1,500 to 3,000 circRNAs passed our cutoffs in most cell types, some cell types (for example, HFDPCs (follicle dermal papilla cells)) had approximately three times more circRNAs in the final catalog than others (for example, HAoECs (thoracic aortic endothelial cells)) (Figure 3C). This variation could not be explained by the differences in sequencing depths (Additional file 6).

Although some circRNAs (including CDR1as) were more ubiquitously expressed, most were found in only a few cell types (Figure 3D). To assess whether circRNAs were any more cell type specific than their linear counterparts, we compared the Jensen-Shannon specificity scores [19] of circRNAs with those of a cohort of linearly spliced exon pairs with the same distribution of expression levels (that is, the same distribution of total junction-spanning reads) as the circRNA set. The expression of circular junctions was not more cell type-specific than that of the control cohort of linear junctions (Figure 3E), and the expression of both was less cell type-specific than that of lincRNAs [19]. To test whether the efficiency of circularization might be regulated in a cell-type-specific manner, we examined the circular fractions of 1,299 circRNAs for which the availability of both the donor and the acceptor sites were each supported by ≥5 reads in all 39 samples. The circular fractions of these circRNAs were nearly as correlated between cell types (median Spearman’s ρ = 0.60 to 0.75) (Figure 3F) as they were between biological replicates (median Spearman’s ρ = 0.75). Taken together, our results suggested that circRNA expression is not any more regulated than expected from the availability of the primary transcripts. We compiled a list of 57 circRNAs, including CDR1as, for which the circular fraction was ≥50% in most cell types in which transcript isoforms were detected (Table S2B in Additional file 3).

To examine their subcellular localization, we quantified the circular fractions of circRNAs in each of the subcellularly fractionated K562 samples, focusing on the 514 circRNAs detected in the K562 whole-cell samples (Additional file 7). Consistent with previous results on a few circRNAs [12,14], most of these circRNAs were predominantly in the poly(A)-depleted cytoplasmic samples.

### Conservation of circRNAs between human and mouse

**Figure 4:**
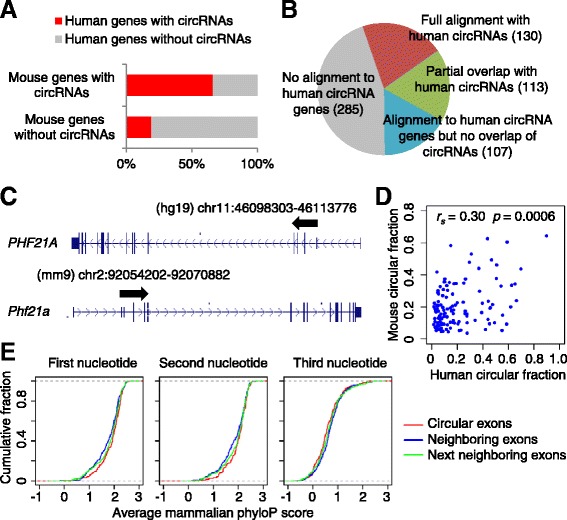
**Conservation between human and mouse circRNAs. (A)** Analysis of enrichment in circRNAs from human orthologs of mouse genes for which circRNAs were found. Only the mouse genes that had one-to-one human orthologs were considered. **(B)** Extent to which mouse circRNAs align with human circRNA loci. **(C)** An example of conserved circRNAs, which derives from human *PHF21A* and mouse *Phf21a* loci. **(D)** Relationship between average circular fractions observed for circRNAs conserved in human and mouse (n = 130). Spearman’s rank correlation coefficient is shown. **(E)** Sequence conservation for the conserved circRNAs, compared with that of their neighboring exons. Distributions are of average mammalian phyloP scores for each of the three codon positions in circular exons and their neighboring linear exons. No significant difference was observed at any of the three positions (*P* > 0.1, paired Mann-Whitney test).

Using the non-poly(A)-selected RNA-seq data from mouse ENCODE cell lines and some other available non-poly(A)-selected RNA-seq datasets (Table S1B in Additional file 1), we also identified and quantified 635 robustly detectable mouse circRNAs (Additional file 8). When analyzing human and mouse genes with clear one-to-one orthologs, we observed that if the mouse gene had a circRNA in our dataset, its human ortholog was likely to also have one (66%), whereas if the mouse gene did not have a circRNA in our dataset, the human gene was less likely to have one (19%) (Figure 4A). The overlap of human and mouse circRNAs genes was not simply due to similarity in exon numbers between orthologs because the enrichment was still observed within subsets of mouse genes grouped by exon numbers (Additional file 9). To test whether human and mouse circRNAs arose from orthologous exons, we used whole-genome alignments to identify the regions of the mouse genome that corresponded to the human circRNAs (no longer limiting the analysis to one-to-one orthologs) and quantified the degree to which our mouse circRNAs overlapped these regions. Among the 350 mouse circRNAs for which the aligned human gene orthologs also had circRNAs, about a third used the orthologous splice sites of human circRNAs (a higher rate than that previously reported [14]), whereas the remaining two-thirds either partially overlapped (32%) or did not overlap (31%) with aligned human circRNA loci (Figure 4B,C). These results indicated that human and mouse circRNAs were often generated not only from orthologous genes but also from orthologous exons. The circular fractions of mouse circRNAs (averaged across all cell types in which the transcript was represented by both donor- and acceptor-matching reads) were weakly yet significantly correlated with those of their human orthologs (Spearman’s ρ = 0.30; Figure 4D), which was slightly lower than those between any two human cell types (typically 0.60 to 0.75).

The derivation of most circRNAs from coding exons complicates analysis of sequence conservation that might provide evidence for sequence-dependent biological function of the circular isoforms. A previous analysis of 223 circRNAs that both derive from coding exons and have orthologous circular isoforms in mouse reported elevated conservation levels in the third nucleotide positions of codons when compared to a control cohort of linear coding exons that were chosen to match the conservation levels at the first and second codon positions [8]. We were able to reproduce these results using the previous list of circRNAs and found that the elevated conservation at the third codon positions was robust when compared with 1,000 different control cohorts (Figure S7A in Additional file 10). Applying this analysis to our list of 130 human circRNAs with mouse orthologs also indicated elevated conservation of the third codon positions (Figure S7A in Additional file 10). Following up on this result, we compared the nucleotide conservation of coding exons within circRNAs to their neighboring linear coding exons, reasoning that the neighboring linear exons would better control for transcript expression levels as well as other unanticipated factors that might correlate with circRNA identification. When using these alternative controls, we did not detect significantly elevated conservation in the third codon positions for either the previous list of circRNAs (Figure S7B in Additional file 10) or our new list (Figure 4E), which argued against the notion that sequence-dependent noncoding functions are enriched within circRNAs.

### No evidence for translation of circRNAs

**Figure 5:**
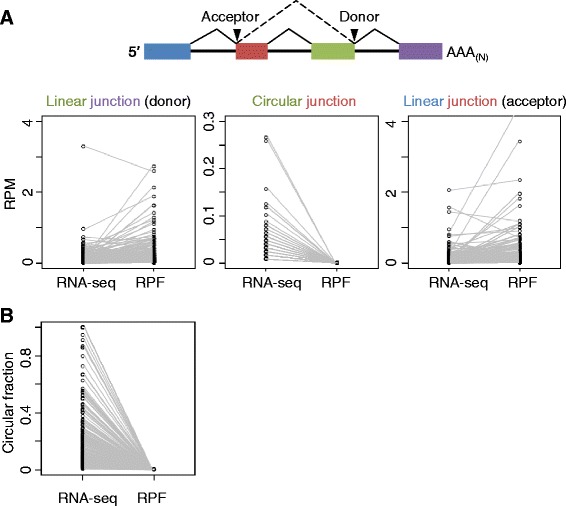
**No evidence for translation of human circRNAs. (A)** Numbers of RNA-seq and RPF reads that spanned the linear junction at the donor end, the circular junction, and the linear junction at the acceptor end of 224 circRNAs that contained RPF reads corresponding to both linear junctions in U2OS cells. **(B)** Circular fractions of 224 of the circRNAs of (A), calculated using either RNA-seq or RPF reads.

The observation that most circRNAs are cytosolic [12] and originate from protein-coding sequences raised the question of whether they could be loaded into the ribosome and be translated into polypeptides. Although circRNAs are devoid of the structures typically required for efficient translation initiation, that is, a 5′ cap and 3′ poly(A) tail, cap-independent translation has been reported for many linear mRNAs [22], and translation can proceed on circRNAs once initiated from an internal ribosome entry site [23]. A few abundant circRNAs have been previously shown to be untranslated [14]. To search systematically for evidence of circRNA translation, we examined both ribosome footprinting data and non-poly(A)-selected RNA-seq data for human U2OS cells. Of the 717 circRNAs with RNA-seq reads spanning their circular junctions, 236 had ribosome protected fragments (RPFs) spanning the RefSeq-annotated linear junctions at both splice sites. Strikingly, after excluding the false-positive junction-spanning reads arising from adjacent paralogous genes (12 instances), no RPF reads could be found spanning any of the remaining 224 circRNA junctions (Figure 5A), which led to uniformly zero circular fractions; that is, every informative RPF corresponded to the linear isoforms (Figure 5B). Making the reasonable assumption that translation in alternative frames (which might terminate prior to reaching the circular junction) is rare, our results showed that, compared with their linear isoforms, most circular isoforms are translated far less efficiently if at all in human U2OS cells. Moreover, because trans-splicing is unlikely to affect translational initiation, the absence of RPFs mapping across the junctions that we classified as circular provided additional evidence that these junctions were indeed circular and not generated by trans-splicing. As more ribosome profiling data become available, it will be interesting to re-visit the question of whether some circRNAs might be translated in other cell types or species.

### The potential of other circRNAs to act as miRNA sponges

**Figure 6:**
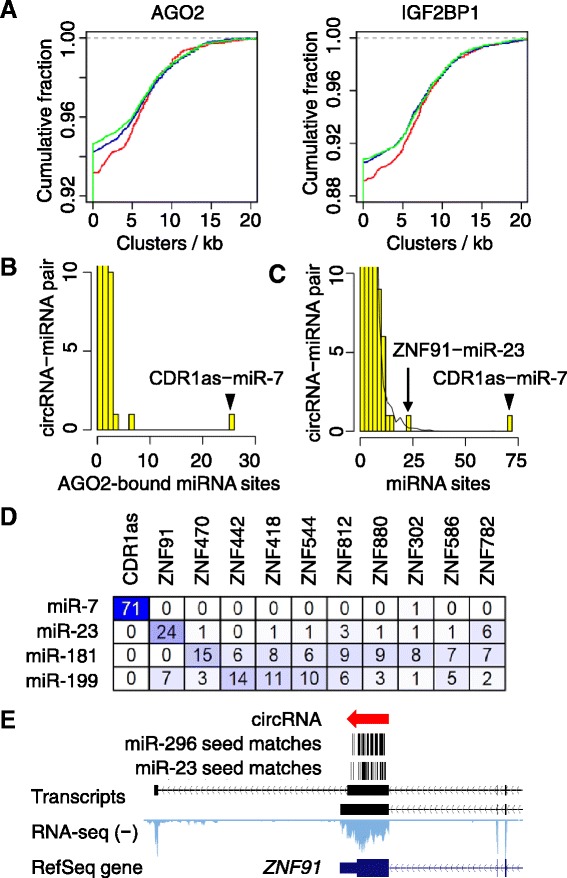
**A search for additional circRNAs with the expected properties of miRNA sponges. (A)** Frequency of AGO2-crosslinking clusters observed in circRNAs compared with that of clusters observed in their neighboring exons (left). See Figure 4E for color keys. For comparison, the analysis was repeated for a negative control, IGF2BP1 (right). No significant difference was observed between circular exons and their neighboring exons (*P* > 0.1, paired Mann-Whitney test). **(B)** Numbers of AGO2-crosslinking clusters assigned to individual miRNA families. The number of crosslinking clusters was tallied for each circRNA-miRNA pair, and the distribution of values is plotted. The outlying CDR1as-miR-7 pair is indicated. **(C)** Numbers of 7- and 8-nucleotide sites for individual miRNA families found within each circRNA. The number of sites was tallied for each circRNA-miRNA pair, and the distribution of values is plotted. The black curve indicates the averaged results when repeating the analysis 1,000 times using different permutations of the site sequences. The two outlying pairs are indicated. **(D)** Numbers of miRNA target sites in CDR1as and top-ranking ZNF circRNAs. **(E)** Part of the *ZNF91* locus containing the circRNA. miR-23 and miR-296 seed matches are indicated.

To search for additional miRNA sponges that resemble CDR1as, we considered several expected properties of strong miRNA sponges. First, miRNA sponges would be expected to bind many miRNA-loaded Argonaute proteins. Using data from high-throughput *in vivo* crosslinking experiments, which identified clusters of AGO2-crosslinking sites that indicated AGO2 binding [24–26], we compared the density of AGO2-crosslinking clusters within exons that can form circRNAs to the density within their neighboring linear exons. Exons that can form circRNAs did not exhibit greater cluster densities for AGO2, with results resembling those for another RNA-binding protein, IGF2BP1 (insulin-related growth factor 2-binding protein 1) (Figure 6A). Similar analyses on 20 additional RNA-binding proteins showed that circular exons generally had slightly higher cluster densities than their neighboring exons (Additional file 11), which could be due to either the circRNAs providing binding sites in addition to those provided by the same exons in linear isoforms, or the lack of translation of circular exons, which would prevent proteins from being displaced by the translocating ribosome. Strikingly, when counting the clusters of AGO2 crosslinks mapping to each circRNA [27], CDR1as had 26 clusters corresponding to miR-7 sites, which was by far the most mapping to any circRNA for any miRNA family (Figure 6B). No other circRNA stood out as a candidate to act as a strong sponge for any of the other RNA-binding proteins.

Because the AGO2-crosslinking sites were determined in HEK293 cells, circRNAs and miRNAs not expressed in HEK293 cells were missed by this analysis. We thus concatenated the annotated exons within each circRNA, and counted the number of canonical 7- and 8-nucleotide target sites [7] for each of the 87 miRNA families conserved across vertebrates. Again, CDR1as ranked on top, containing 71 miR-7 sites (Figure 6C). CDR1as-miR-7 was also the only circRNA-miRNA pair that exceeded the upper limit of results from the negative control, in which the analysis was repeated with permutated miRNA sequences (Figure 6C). We conclude that among the human circRNAs, CDR1as stands alone as the most compelling miRNA sponge for any conserved miRNA seed family.

Our analysis of miRNA site number also pointed to circRNAs from the repeat-rich C_2_H_2_ zinc finger (ZNF) gene family (Figure 6D). In particular, a circRNA generated from the *ZNF91* locus (circRNA-ZNF91) contains 24 miR-23 sites (Figure 6E), 19 of which were 8-nucleotide sites. These numbers exceeded that of the other proposed miRNA sponge, mouse *Sry*, which has 16 miR-138 sites [9]. *ZNF91* belongs to a C_2_H_2_ zinc finger (ZNF) gene family that is greatly expanded in the primate lineage and known to contain exceptionally abundant target sites for several miRNA families, including miR-23, miR-181 and miR-199 [28]. The next nine ZNF circRNAs ranked by the total number of sites for these three miRNA families had 7 to 15 sites to one of the 3 families (Figure 6D). Expanding our miRNA site search beyond the 87 miRNA families conserved beyond mammals to the 66 miRNA families conserved only within the mammalian lineage (Figure S9A in Additional file 12), we found that circRNA-ZNF91 had 39 additional sites for miR-296 (Figure 6E). CDR1as also had 22 sites for the miR-876-5p/3167 family (Figure S9B in Additional file 12), although they were not as conserved as the miR-7 sites.

## Discussion

Because molecular studies of eukaryotic RNA typically begin with poly(A)-selection, circRNAs have often escaped detection and consideration. Our study adds to previous circRNA annotation efforts [8,12,14,15] to yield an expanded catalog of circRNAs robustly detected from a large variety of human cell types. Our circRNA identification method resembles that previously used [8,14], except we focused our analyses on the circRNA loci with circular fractions ≥10%. Other recent studies take a more targeted approach and search for back-spliced junctions from annotated splice sites [12,15] and therefore miss the unannotated genes and exons, especially those that have particularly high circular fractions and are rarely found in the poly(A)^+^ RNA-seq data, such as CDR1as. Moreover, unlike previous studies that identify circRNAs from poly(A)-depleted RNA-seq data [14,15], we applied our pipeline to non-poly(A)-selected RNA-seq data, which were neither depleted nor enriched in circRNAs or their linear isoforms. An advantage of using these datasets is that we could directly estimate circular fractions without experimental calibration [15].

With this catalog of 7,112 human circRNAs in hand, the key question is whether they comprise an underappreciated class of molecules with cellular functions, or whether they are largely inert side-products of imperfect pre-mRNA splicing. The circRNA with the most compelling evidence for a biological function is the miR-7 sponge, CDR1as. Although a biological context has not yet been identified in which CDR1as loss-of-function influences miR-7 activity, this circRNA has >60 conserved sites to miR-7 and a developmental phenotype following its ectopic delivery [8,9]. The other circRNA proposed to act as a miRNA sponge, mouse *Sry* [9], has only one miR-138 site in its human homolog, which indicates that the proposed sponge function is not conserved in mammals.

What about functional potential of the other 7,000-plus circRNAs? By characterizing the molecular abundance and translation of circRNAs and providing an updated perspective on their sequence conservation and potential to act as miRNA sponges, our analyses can speak to this question. Although we found thousands of circRNAs in each cell type, only approximately 2% (20 to 60, depending on the cell type) had circular fractions exceeding 50%, which indicates that most were minor alternative isoforms of their respective primary transcripts. Moreover, fewer than 10% had FPKMs ≥10 in any of the 39 samples examined. Considering that in homogeneous cell types one molecule per cell usually corresponds to an FPKM of 1 to 4 [29], most circRNAs only accumulated to a few molecules per cell. This generally low circular fraction and weak accumulation was observed despite the expectation that each circRNA, by virtue of its exonuclease insusceptibility, might persist in the cell much longer than its linear alternative isoforms. Such low accumulation would not be expected of molecules that titrate miRNAs or other abundant regulators away from their regulatory targets. Indeed, we find few circRNAs with the properties expected of miRNA sponges. When circRNAs are experimentally enriched by either poly(A)-depletion [15] or RNase R digestion [14], tens of thousands of more circRNAs are found, even when limiting the search to only those that use annotated splice sites. Many of these low-abundance circRNAs have zero junction-spanning reads when we searched in the non-poly(A)-selected RNA-seq data, in which circRNAs were neither enriched nor depleted (Additional file 5). Perhaps it is not too far-fetched to speculate that all multi-exon genes generate one or more circular isoforms at low frequencies, whereas circularization of CDR1as is specific and efficient in all cell types in which it is expressed.

To have a physiological effect at such low levels, circRNAs would need to either participate in a catalytic process or interact very specifically with other molecules that have important functions when present at very low cellular levels. For example, mRNAs have physiological effects when present at only a few molecules per cell because they participate in the catalytic process of translation, which can produce many protein molecules from each mRNA molecule. However, we found that circRNAs are rarely translated. Some linear lincRNAs are proposed to interact with and modulate the output of a single genomic locus, which would explain their physiological effect despite their relatively low cellular abundance [5]. Likewise, a rare circRNA could conceivably recognize and regulate a rare mRNA. However, a specific, high-affinity interaction with an mRNA or other rare cellular component would presumably rely on the circRNA sequence, which would need to be conserved to retain its function over evolutionary time, yet we found no evidence for circRNA sequence conservation beyond that observed for neighboring linear exons.

We suspect that CDRas is not the only circRNA with an evolutionarily conserved biological function. This being said, our observations that most circRNAs 1) are inefficiently produced relative to their linear alternative isoforms, 2) accumulate to only low levels in the cell, and 3) are no more conserved than their neighboring linear exons, when considered together, suggest that most circRNAs may be inconsequential side-products of imperfect pre-mRNA splicing. For linear alternative-spliced isoforms, preferential production of orthologous isoforms in the same tissues of different species is considered evidence of function [30,31]. For circular isoforms, this type of analysis would require non-poly(A)-selected datasets from the same tissues of different species, which unfortunately are not yet available. For now, the only observation consistent with the idea that many circRNAs could be functional is our finding that the loci that produce circRNAs in mouse also tend to do so in humans. However, retention of circRNA production since the last common ancestor of mouse and human could have other causes apart from selection for circRNA function. For example, slowed splicing at the circRNA acceptor would presumably favor circRNA production because it would allow for transcription of the downstream donor, and if this slowed splicing is conserved for reasons other than circRNA function, then the production of circRNAs might nonetheless be conserved. Therefore, considering the conserved production of circRNAs as evidence against the idea that the vast majority of circRNAs are inert splicing side-products would require a more thorough understanding of the determinants of circRNA biogenesis.

## Conclusions

Mammalian cells produce a large number of circRNAs, which have captured the interest of many biologists, particularly after the description of CDR1as and its many conserved sites to miR-7. Our work identifies thousands of additional circRNAs and focuses on those that have circular fractions ≥10%. Unlike CDR1as, most of the previously and newly identified mammalian circRNAs represent alternatively spliced, low-abundance isoforms of protein-coding genes. Expression of circRNAs is generally not more cell-type-specific than mRNAs with similar overall expression levels. Although orthologous circRNAs were found between mouse and human, their sequence conservation is no higher than that of their neighboring linear exons, and no other identified circRNA is expected to function as a miRNA sponge nearly as effectively as CDR1as. Although some circRNAs with biological functions might exist, our results suggest that a large majority of circRNAs are inconsequential side-products of pre-mRNA splicing.

## Materials and methods

### circRNA identification and quantification

Human and mouse Ribo-Zero RNA-seq data were downloaded from either the ENCODE project or Gene Expression Omnibus (GEO). For each sample, Fastq reads were first mapped to hg19 or mm9 genome by Bowtie, allowing 2 mismatches. After removing PCR-duplicated reads by FASTX toolkit, all the unmapped reads were then aligned by BLAT (no mismatch or gap allowed). Dual alignments of two complimentary segments within a single read mapping to two regions on the same chromosome in the reverse order and no more than 100 kb away from each other were selected as circular-junction candidates. Next, GT and AG dinucleotides were searched for within 10 nucleotides genomic windows flanking the donor and acceptor end of each junction, respectively. Candidates with GT-AG-flanking junctions were carried forward, and the GT-AG dinucleotides were used to identify the precise splice sites. For human circRNAs, each junction required support from at least two independent reads within the sample.

To quantify the relative ratio of circular and linear isoforms, we focused on the two segments (20 nucleotides upstream from the donor and 20 nucleotides downstream from the acceptor) flanking the circular junction. Because many linear isoforms may exist for a given splice site, we took an inclusive approach and simply counted the reads that contained either of these two sequences and have enough sequence space for the other sequence (*n*_donor_ and *n*_acceptor_), and the reads that spanned the circular junction and contained both sequences (*n*_junction_). The circular fraction is calculated as *n*_junction_ / (*n*_donor_ + *n*_acceptor_ – *n*_junction_ + 1). To be accepted into the final circRNA catalog, a circRNA candidate must have a circular fraction ≥ 10% in at least two samples.

### Conservation analyses

One-to-one gene ortholog tables for gene-level analysis were downloaded from Ensembl [32]. For exon-level analysis, human circRNA junction coordinates were converted to mouse (mm9) genome coordinates using the UCSC liftOver tool, then intersected with mouse circRNA junctions using BEDTools. To calculate the correlation of average circular fractions of circRNA orthologs, circular fractions of each circRNA in all cell types wherein it was expressed (≥1 read for each of the donor and acceptor ends) were averaged. Spearman’s rank correlation test was performed.

### Analysis of translation

Twenty-nucleotide sequences were taken from circular junctions and each of the two linear junctions overlapping the circular junctions (10 nucleotides from each side of each junction). Numbers of reads containing each of these sequences, as well as the circular fractions for each circRNA, were compared using RNA-seq and RPF data from human U2OS cells.

### miRNA and protein binding sites

PAR-CLIP data were downloaded from the GEO. After read alignment by Bowtie, binding clusters were identified using PARalyzer with default settings [24]. Cluster densities of all circular exons were calculated and compared to those of their linear neighboring exons. To avoid biases, only coding exons were considered. To quantify miRNA targets sites, exonic segments within each circRNA were concatenated using the transcript models built from all ENCODE cytosolic RNA-seq data, and numbers of canonical miRNA sites (7mer-A1, 7mer-m8, and 8mer sites) [7] for the 87 miRNA families conserved across vertebrates and 66 miRNA families conserved across mammals were quantified for each circRNA. To estimate the distribution of sites expected by chance, the procedure was repeated using 1,000 cohorts consisting of 87 or 66 control *k*-mers. To select a control *k*-mer, each 8mer site was randomly permuted to preserve its mononucleotide composition. Permutated sequences were chosen if they preserved the CG dinucleotide number and possessed an A at the 3′-most nucleotide. Collectively, these constraints served to select control *k*-mers with similar expected genome-wide abundance.

### Data availability

RNA-seq and RPF data of human U2OS cells have been deposited in GEO under accession number GSE51584.

## Additional files

Additional file 1: Table S1. Non-poly(A)-selected RNA-seq data used in this study. Additional file 2: Figure S1. Sequence characteristics of circular junctions. Additional file 3: Table S2. Human circRNA catalog. Additional file 4: Figure S2. Length distribution of circRNAs. Additional file 5: Figure S3. Comparison between circRNA annotations. Additional file 6: Figure S4. Relationship between number of circRNAs detected in each sample and sequencing depth. Additional file 7: Figure S5. Subcellular localization of circRNAs in K562 cells. Additional file 8: Table S3. Mouse circRNA catalog. Additional file 9: Figure S6. Enrichment in circRNAs from human orthologs of mouse genes for which circRNAs were found. Additional file 10: Figure S7. Protein-coding-independent conservation of circRNAs. Additional file 11: Figure S8. Frequency of crosslinking clusters observed in circRNAs compared to that of clusters observed in their neighboring exons. Additional file 12: Figure S9. Sites for mammal-specific miRNA families found within each circRNA.

## Abbreviations

circRNA: circular RNA
FPKM: fragments per kilobase of transcript per million fragments sequenced
GEO: Gene Expression Omnibus
lincRNA: long intervening non-coding RNA
miRNA: microRNA
ncRNA: non-protein-coding RNA
RPF: ribosome protected fragment
UTR: untranslated region
ZNF: zinc finger
