# Supplementary material for: Expanded identification and characterization of mammalian circular RNAs
Source: Genome Biol. 2014 Jul 29;15(7):409. doi: 10.1186/s13059-014-0409-z (PMC4165365; doi:10.1186/s13059-014-0409-z)
Supplement: Supplementary file 1 [file 13059_2014_409_MOESM10_ESM.doc]

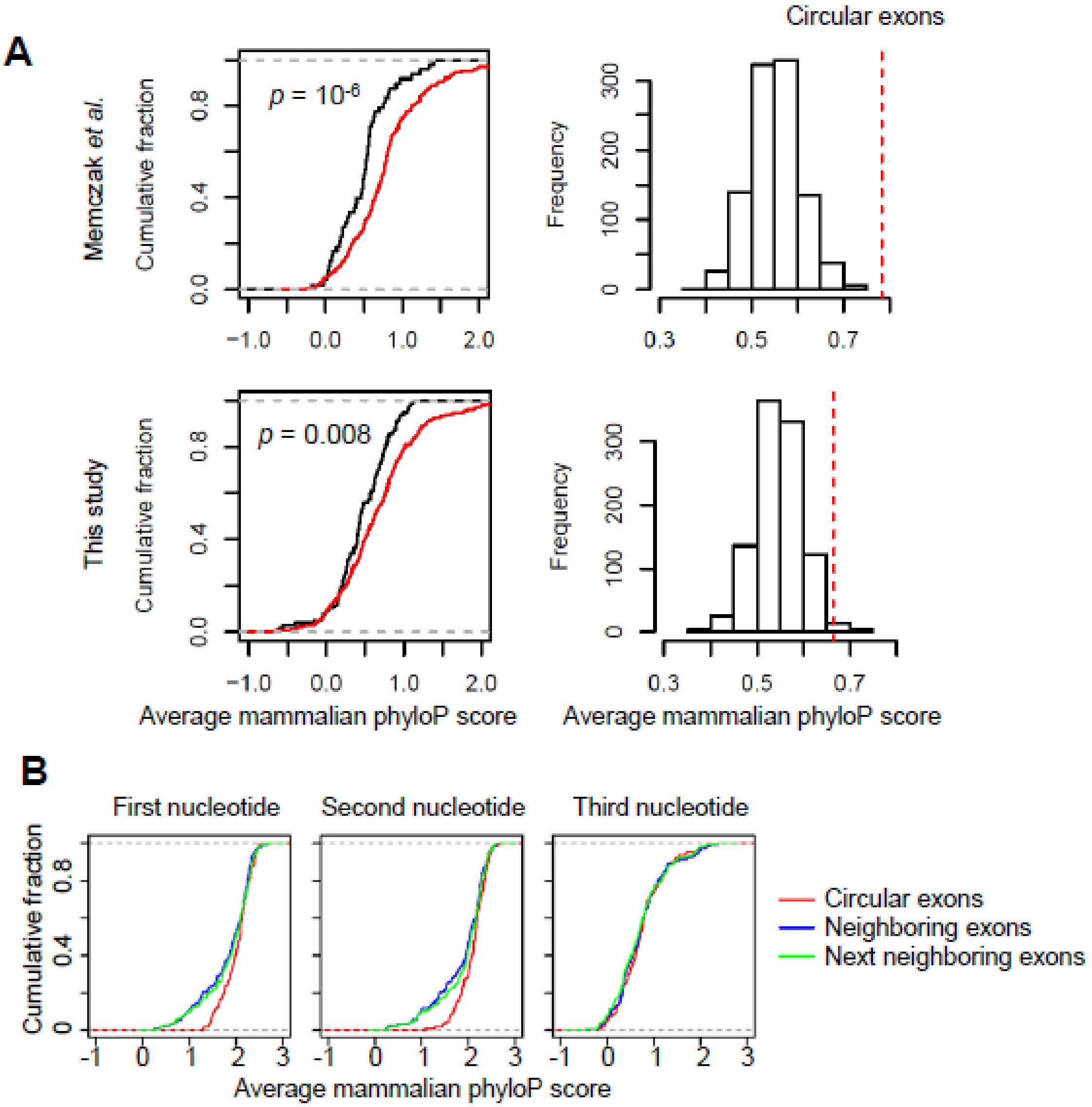

Supplemental Figure S7 Protein-coding-independent conservation of circRNAs. (A) Third-nucleotide conservation of coding sequences within circular exons previously reported to be conserved in mouse (Memczak *et al.*, *top*) and the conserved circular exons identified in this study (*bottom*). Each circular exon was compared to randomly chosen first- and second-nucleotide conservation-matched linear exons. Cumulative functions of the third nucleotide phyloP scores of circRNAs (red) and one control group (black) are shown on the left (*p* values, Kolmogorov–Smirnov tests). Shown on the right are the histograms of the means of phyloP scores of 1,000 control groups. The mean of the third nucleotide phyloP scores of circular exons are indicated by the dashed red line. **(B)** Similar to Fig. 3E, except analysis is of the circular exons previously reported to be conserved in mouse (Memczak *et al.*). Shown are cumulative functions of the first-, second- and third-nucleotide phyloP scores of conserved circRNAs and those of their linear neighboring exons.
