# Supplementary material for: Expanded identification and characterization of mammalian circular RNAs
Source: Genome Biol. 2014 Jul 29;15(7):409. doi: 10.1186/s13059-014-0409-z (PMC4165365; doi:10.1186/s13059-014-0409-z)
Supplement: Supplementary file 2 [file 13059_2014_409_MOESM11_ESM.doc]

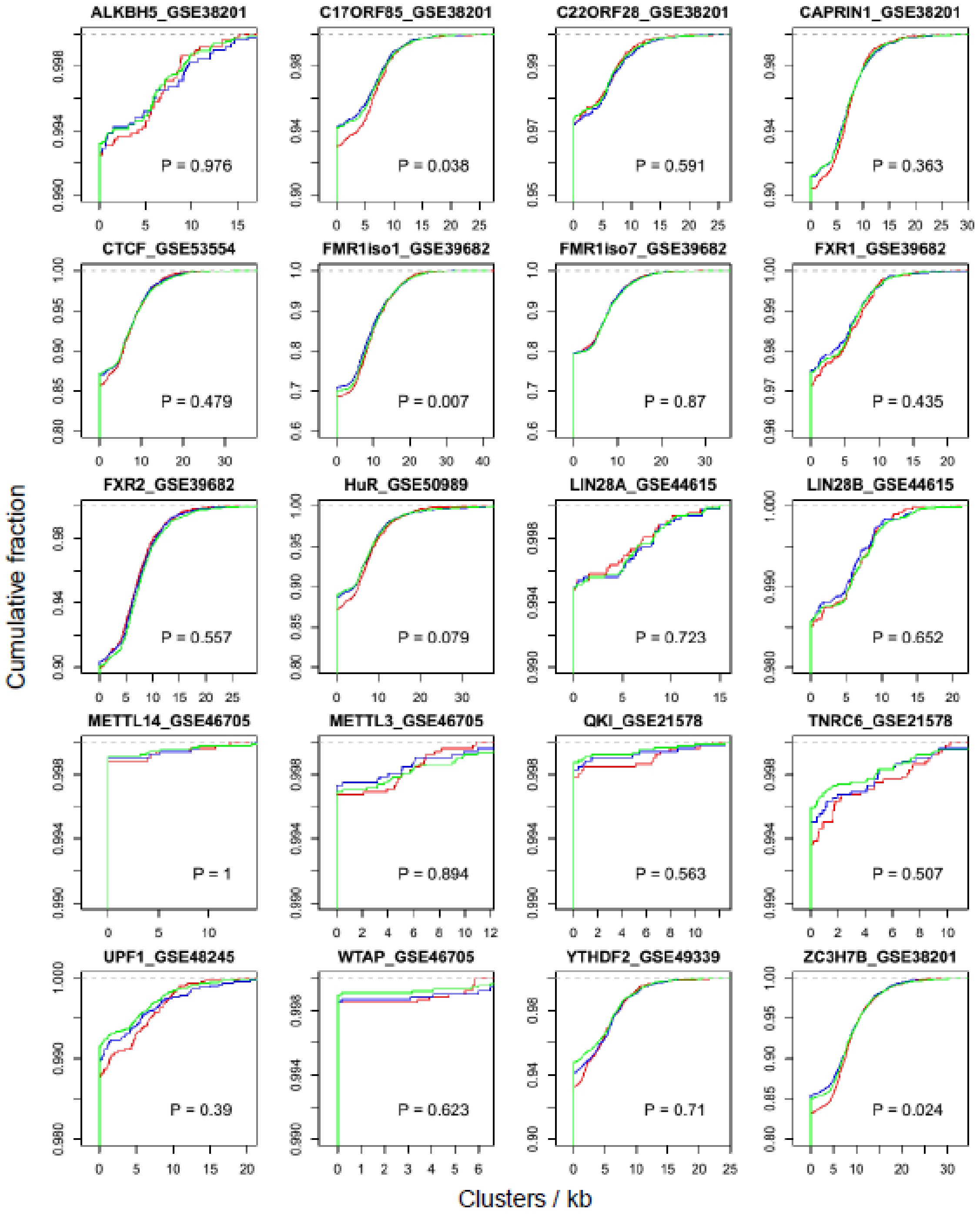

Supplemental Figure S8 Frequency of crosslinking clusters observed in circRNAs compared to that of clusters observed in their neighboring exons. See Fig. 4E for color keys. P values of paired Mann-Whitney tests between circular exons and their neighboring exons are shown.
