# Supplementary material for: Expanded identification and characterization of mammalian circular RNAs
Source: Genome Biol. 2014 Jul 29;15(7):409. doi: 10.1186/s13059-014-0409-z (PMC4165365; doi:10.1186/s13059-014-0409-z)
Supplement: Supplementary file 3 [file 13059_2014_409_MOESM12_ESM.doc]

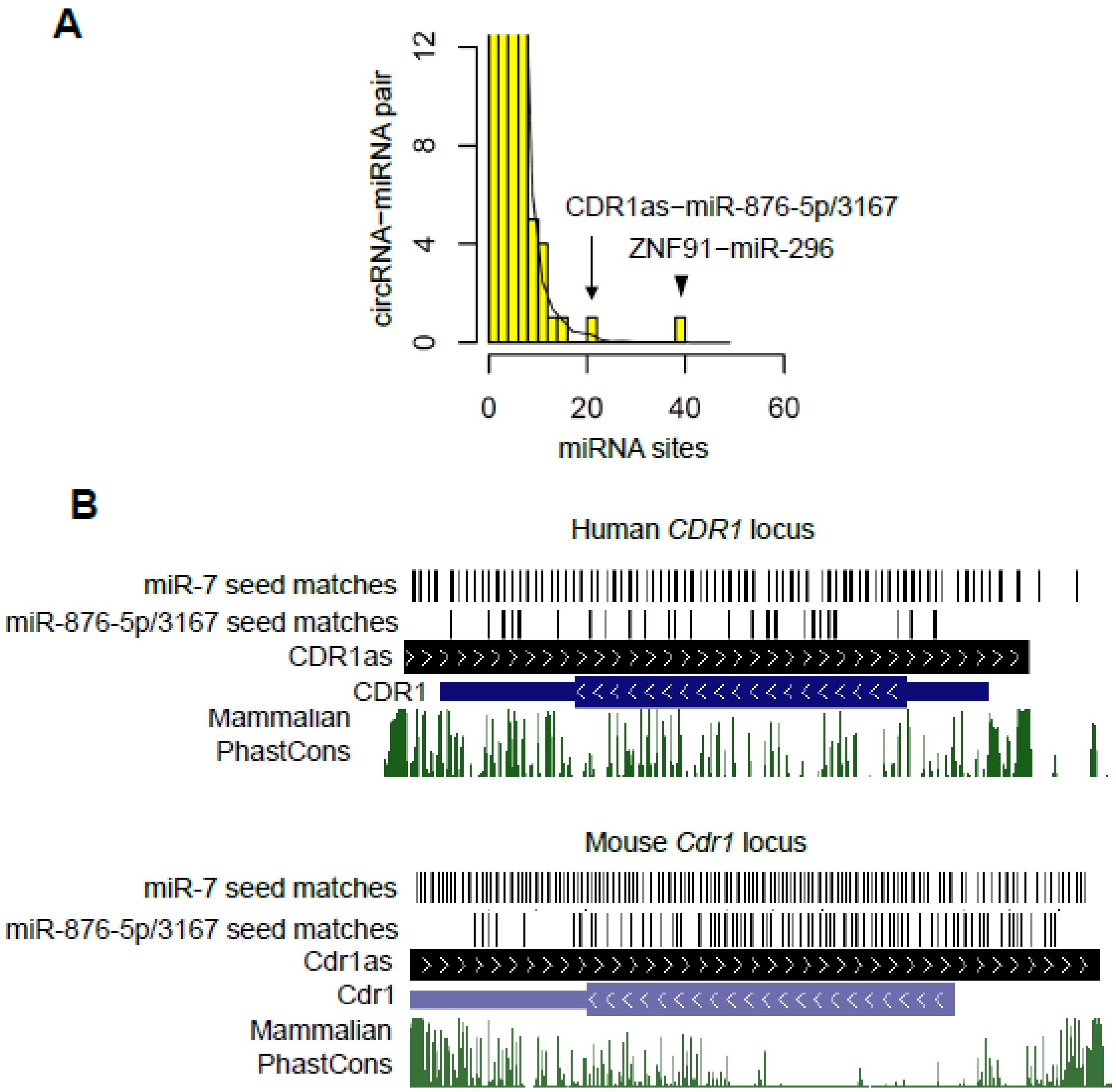

Supplemental Figure S9 Sites for mammal-specific miRNA families found within each circRNA. (A) Similarly to Fig.6C, the number of sites was tallied for each circRNA–miRNA pair, and the distribution of values is plotted. The black curve indicates the averaged results when repeating the analysis 1,000 times using different permutations of the site sequences. The two outlying pairs are indicated. **(B)** The human *CDR1* (*top*) and mouse *Cdr1* (*bottom*) loci. miR-7 and miR-876-5p/3167 seed matches are indicated.
