# Supplementary material for: Expanded identification and characterization of mammalian circular RNAs
Source: Genome Biol. 2014 Jul 29;15(7):409. doi: 10.1186/s13059-014-0409-z (PMC4165365; doi:10.1186/s13059-014-0409-z)
Supplement: Supplementary file 4 [file 13059_2014_409_MOESM2_ESM.doc]

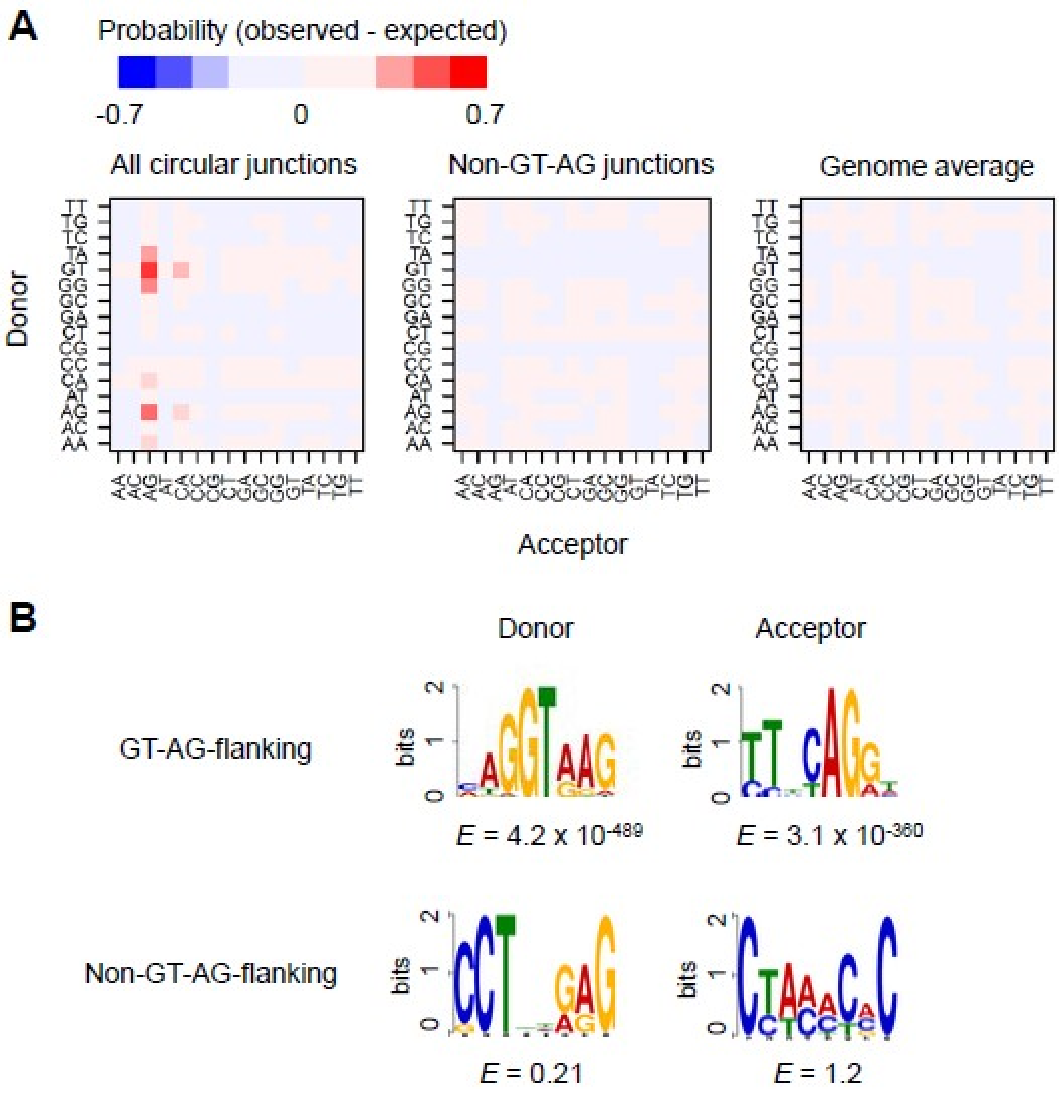

Supplemental Figure S1 Sequence characteristics of circular junctions
(A) Enrichment of each combination of dinucleotides in the 10 nt windows flanking the donor and acceptor sites of all the circular junctions (*left*) and non-GT-AG-flanking junctions (*middle*). The expected probability for each combination was calculated from shuffled sequences that preserved the single nucleotide composition. No dinucleotide was enriched in randomly sampled genomic windows (*right*). **(B)** Enriched motifs flanking the donor (*left*) and acceptor (*right*) sites of GT-AG-flanking (*top*) and non-GT-AG-flanking circular junctions (*bottom*). *E* values calculated by MEME are shown.
