# Supplementary material for: Expanded identification and characterization of mammalian circular RNAs
Source: Genome Biol. 2014 Jul 29;15(7):409. doi: 10.1186/s13059-014-0409-z (PMC4165365; doi:10.1186/s13059-014-0409-z)
Supplement: Supplementary file 5 [file 13059_2014_409_MOESM4_ESM.doc]

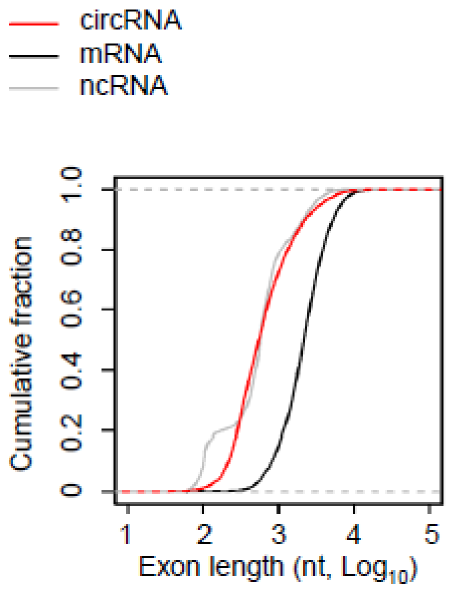

Supplemental Figure S2 Length distribution of circRNAs. The exonic sequence lengths of all circRNAs were compared to those of GENCODE-annotated mRNAs and ncRNAs.
