# Supplementary material for: Expanded identification and characterization of mammalian circular RNAs
Source: Genome Biol. 2014 Jul 29;15(7):409. doi: 10.1186/s13059-014-0409-z (PMC4165365; doi:10.1186/s13059-014-0409-z)
Supplement: Supplementary file 6 [file 13059_2014_409_MOESM5_ESM.doc]

A

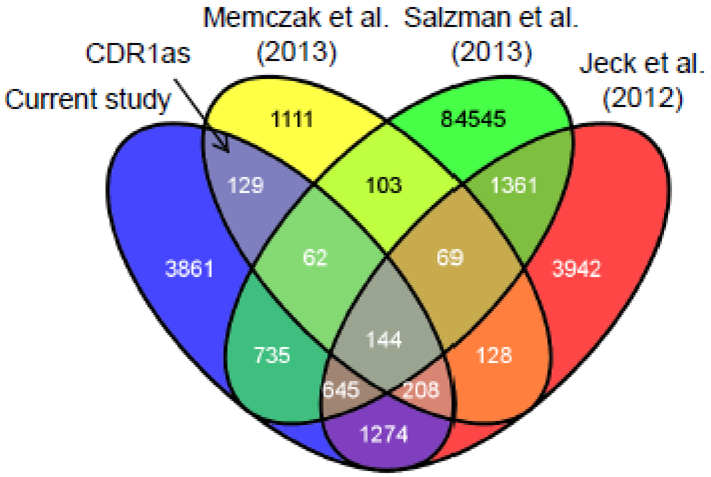

B

Study	Cell type	N	Non-poly(A)-selected RNA-seq data
Current study	CD34+ hematopoietic progenitors	822	SRR534325
Memczak et al. (2013)	CD19+ naïve B cells	1302	SRR364679, SRR384963
Jeck et al. (2012)	Hs68 fibroblasts	1942*	SRR444655, SRR444975
Salzman et al. (2013)	Gm12878 fibroblasts	1535**	SRR089332, SRR089333***

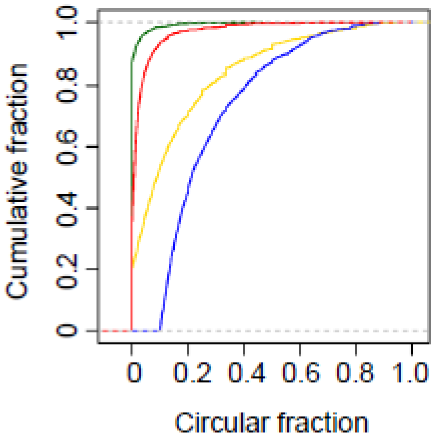

* A random subset of circRNAs were quantified.

** The most highly expressed (based on expression ranking in that study) circRNAs were quantified.

*** Different data from the one used in the study.

Supplemental Figure S3. Comparison between circRNA annotations. (A) A Venn diagram shows the overlap between the total circRNA catalogs from four studies. (B) Circular fractions of circRNAs from the four studies were quantified using Ribo-minus RNA-seq data of the cell types in which these circRNAs were found.
