# Supplementary material for: Expanded identification and characterization of mammalian circular RNAs
Source: Genome Biol. 2014 Jul 29;15(7):409. doi: 10.1186/s13059-014-0409-z (PMC4165365; doi:10.1186/s13059-014-0409-z)
Supplement: Supplementary file 7 [file 13059_2014_409_MOESM6_ESM.doc]

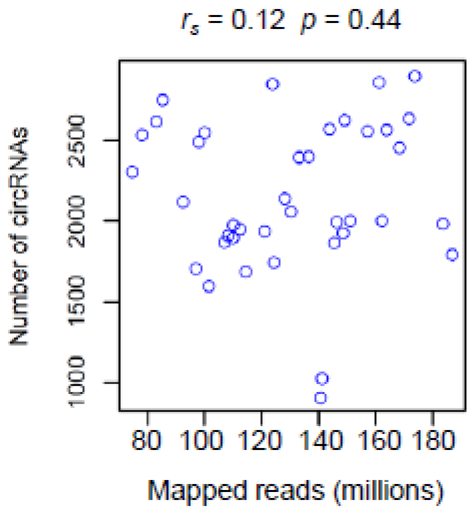

Supplemental Figure S4 Relationship between number of circRNAs detected in each sample and sequencing depth. The total numbers of robustly detected circRNAs in each sample (Fig. 2A) are plotted against the total numbers of Bowtie-mapped reads (read 1; two mismatches allowed). Spearman's rank correlation and p value are shown.
