# Supplementary material for: Expanded identification and characterization of mammalian circular RNAs
Source: Genome Biol. 2014 Jul 29;15(7):409. doi: 10.1186/s13059-014-0409-z (PMC4165365; doi:10.1186/s13059-014-0409-z)
Supplement: Supplementary file 8 [file 13059_2014_409_MOESM7_ESM.doc]

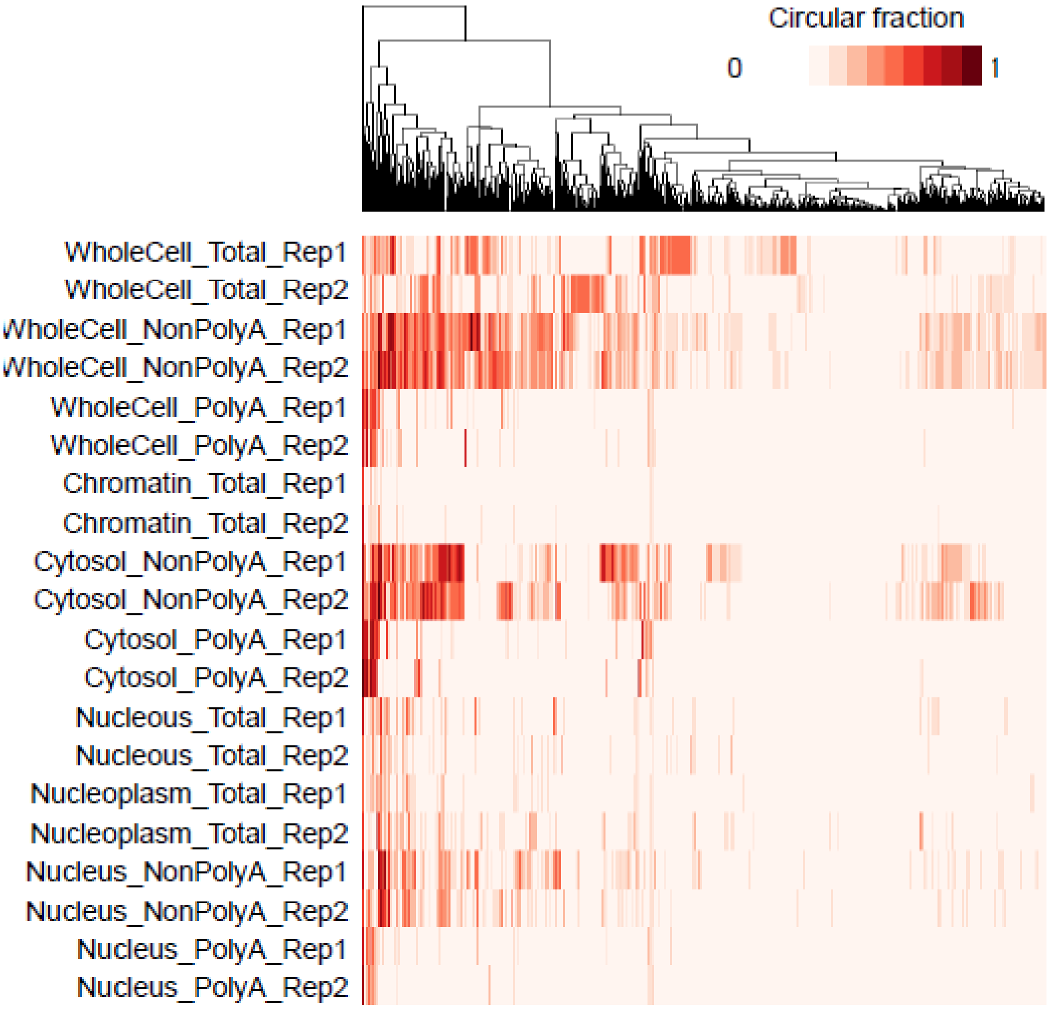

Supplemental Figure S5 Subcellular localization of circRNAs in K562 cells. Unsupervised hierarchical clustering of circular fractions in the indicated ENCODE samples, focusing on of 514 circRNAs detected in the whole-cell samples from K562.
