# Supplementary material for: Expanded identification and characterization of mammalian circular RNAs
Source: Genome Biol. 2014 Jul 29;15(7):409. doi: 10.1186/s13059-014-0409-z (PMC4165365; doi:10.1186/s13059-014-0409-z)
Supplement: Supplementary file 9 [file 13059_2014_409_MOESM9_ESM.doc]

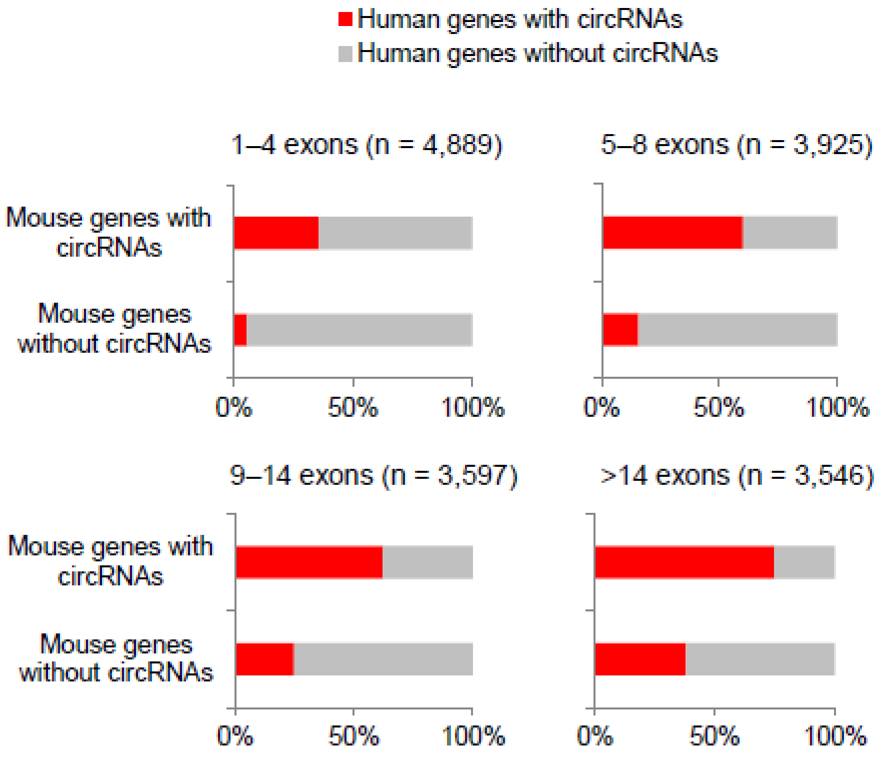

Supplemental Figure S6 Enrichment in circRNAs from human orthologs of mouse genes for which circRNAs were found. Similar to Fig. 3A, except that the genes that had one-to-one orthologs were first grouped into four bins based on the number of exons in the mouse gene. In each bin, the human orthologs of mouse genes that hosted circRNAs were more likely to have circular isoforms than were the human orthologs of mouse genes in which circRNAs were not found. (p <0.001, χ^2 test).
